# Patient Similarity: Emerging Concepts in Systems and Precision Medicine

**DOI:** 10.3389/fphys.2016.00561

**Published:** 2016-11-24

**Authors:** Sherry-Ann Brown

**Affiliations:** Department of Cardiovascular Diseases, Mayo ClinicRochester, MN, USA

**Keywords:** patient similarity, patient similarity analytics, computational medicine, big data analytics, clinical decision support

## Introduction

**Figure 1:**
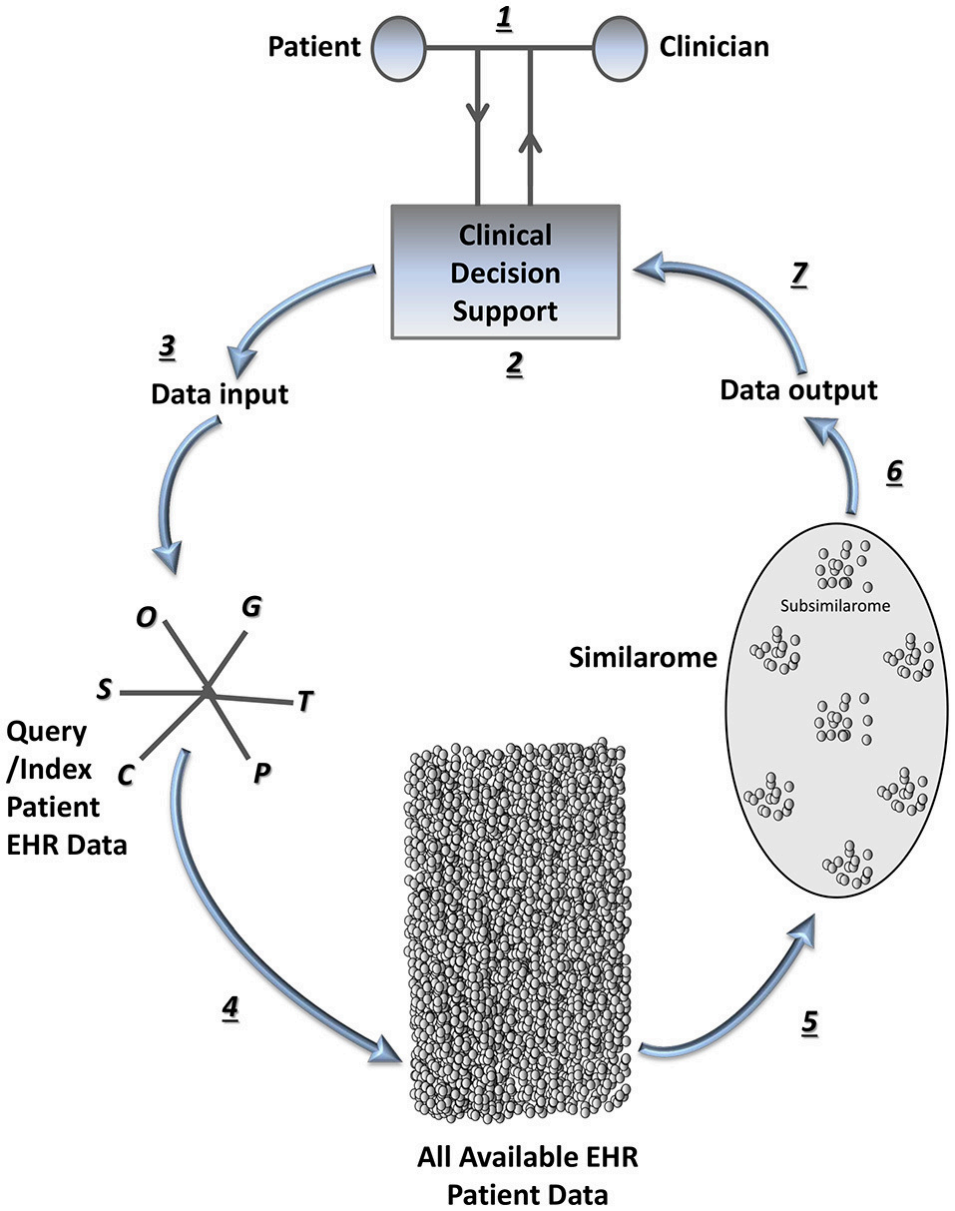
**The patient similarity analytics loop in systems medicine**. Once a query patient is selected, the patient and clinician (e.g., physician or other health professional) in partnership can enter the “patient similarity analytics loop” (step 1), which is iterative as patient characteristics evolve over time and new patients become available for inclusion in the similarome. In step 2, query information is entered via a clinical decision support tool interface. In step 3, this information combines with data from the query or index patient's EHR to form the data input for the patient similarity algorithms. Each “omic” or systems medicine data type or tool (Brown, 2015b) functions as a predictor variable vector, all of which are incorporated into the multidimensional feature space for the patient. In step 4, the entire available EHR patient populous is interrogated with a patient similarity network analysis tool; efficient data mining is completed using patient similarity algorithms. In step 5, similarity data is arranged, yielding a similarome (cohort of patients most similar to the query/index patient), with subsimilaromes (subgroups of patients most similar to the query/index patient based on prioritizing various comorbidities/medications, etc.). Step 6 involves data collating and information retrieval. In step 7, the similarome (which includes subsimilaromes) is presented to the patient-clinician partnership via the clinical decision support tool interface for clinical decision-making at the point-of-care. C, Clinical information; G, Genomics; O, Other systems medicine data types or tools; P, Proteomics; S, Social network data; T, Transcriptomics.

Healthcare data generates a huge *volume* of information in *various* formats at high *velocity* with sometimes questionable *veracity* (Barkhordari and Niamanesh, 2015) (4V). As a result, big data tools such as patient similarity are necessary to facilitate analytics, which reduces costs (Srinivasan and Arunasalam, 2013) and improves healthcare systems (Jee and Kim, 2013). Patient similarity investigates distances between a variety of components of patient data, and determines methods of clustering patients, based on short distances between some of their characteristics. Although patient similarity is in its early stages, ultimately information about diseases, risk factors, lifestyle habits, medication use, co-morbidities, molecular and histopathological information, hospitalizations, or death are compared with laboratory investigations, imaging, and other clinical data assessing medical evidence of human behavior (Figure 1). Such analytics consist of efficient computational analyses with patient stratification by multiple co-occurrence statistics, based on clinical characteristics. Algorithms create subgroups of patients based on similarities among their electronic avatars. Among electronic avatars found to be similar, subgroups of patients can be evaluated by further stratification guided by individual diagnoses, risk factors, medications, and so on. Because of the multiple networks of subgroups of patients, patient similarity can be considered an application of network medicine, with the output termed “patient similarity networks.” Thus, data mining extracts clinically relevant information hidden in clinical notes and embedded in other areas of the electronic health record (EHR) coupled with International Classification of Disease codes. The result is a systematic individualized analysis of a subset of patients that can improve outcome prediction and help guide management for a particular patient currently being cared for by a clinician (Lee et al., 2015). The communication or output from the algorithms can be used to identify and predict disease correlations and occurrence, and potentially for clinical decision support at the point of care. Patient similarity analytics are not restricted to global findings from large clinical trials consisting of somewhat heterogeneous patient populations (Roque et al., 2011). In this way, patient similarity represents a paradigm shift that introduces disruptive innovation to optimize personalization of patient care. Some promising examples are regarding mental and behavioral disorders (Roque et al., 2011), infectious diseases (Li et al., 2015), cancers (Wu et al., 2005; Teng et al., 2007; Chan et al., 2010, 2015; Klenk et al., 2010; Cho and Przytycka, 2013; Li et al., 2015; Wang, 2015; Bolouri et al., 2016; Wang et al., 2016), endocrine (Li et al., 2015; Wang, 2015), and metabolic diseases (Zhang et al., 2014; Ng et al., 2015). Others involve diseases of the nervous system (Lieberman et al., 2005; Carreiro et al., 2013; Cho and Przytycka, 2013; Qian et al., 2014; Buske et al., 2015a; Li et al., 2015; Bolouri et al., 2016; Wang et al., 2016), eyes (Buske et al., 2015a; Li et al., 2015), skin (Buske et al., 2015a; Li et al., 2015), heart (Wu et al., 2005; Tsymbal et al., 2007; Syed and Guttag, 2011; Buske et al., 2015a; Li et al., 2015; Panahiazar et al., 2015a,b; Wang, 2015; Björnson et al., 2016), liver (Chan et al., 2015), intestines (Buske et al., 2015a), musculoskeletal system (Buske et al., 2015a), congenital malformations (Buske et al., 2015a), and various other conditions or factors influencing health status (Gotz et al., 2012; Subirats et al., 2012; Ng et al., 2015).

## Patient similarity in systems medicine

Patient similarity is just starting to spread its wings and has the potential to transform Systems Medicine, which is Systems Biology applied to health care. Systems Biology studies the characteristics of cells, tissues, organisms, or other comprehensive biological units as whole systems. Systems Biology seeks to determine how changes in one part of the system can affect the behavior of the whole system, and often focuses on predictive modeling of the system in a perturbed state. Patient similarity analytics could be developed to bring together characteristics of the patient as a whole human system, and compare these to a multitude of similar patients. Accordingly, patient similarity analytics should in the near future incorporate genomics, transcriptomics, proteomics, microbiomics, and other “omics” and diverse components of systems medicine. In addition, simulation of physiology at the level of the molecule, cell, tissue, organ, and organism should be consolidated as a comprehensive similarity feature to give a broader view of interactions among organ systems. Patient similarity analytics could provide predictive models of a patient's outcome in the setting of disease perturbations or diagnoses relevant to the index patient. Making adjustments in the query data that serve as input for the predictive models would allow for assessment of how new diagnoses or therapies could impact the overall behavior and phenotype of the whole patient.

Beyond the reasoning above, integrating the majority of these systems medicine tools into patient similarity analytics is potentially the next frontier in Systems Medicine, for at least a few reasons. First, patient similarity analytics embrace a systems view by assessing a myriad of characteristics for hundreds or thousands of patients to produce a meaningful and useful result. Second, patient similarity analytics are analogous to various “omics” that in part compose Systems Biology. Just as transcriptomics refers to generation of messenger RNA expression profiles (Briefing, 1999), one could consider a term similaromics referring to generation or identification of patients similar to an index patient. Similaromics is also akin to phenomics, proteomics, and genomics, among others. Phenomics refers to cataloging the observable characteristics conferred by a gene and proteomics describes the generation of proteins expressed by a cell (Briefing, 1999). One might argue that patient similarity is not quite analogous to genomics, since an individual's genome is thought to be constant throughout their lifetime. However, this is no longer necessarily the case, due to the current progress of genome editing tools. Indeed, patient similarity is analogous to these various omics, all with the potential to change over the lifetime of the individual. Thus, just as a genome is the complement of all DNA within a cell, a similarome is the complement of patients found to be similar to an index patient. Within the similarome, one can further distinguish subgroups of patients that are most similar to an index patient, based on preferentially assigning preeminence to comorbidities or medications of most interest or relevance to the index patient, e.g., during a focused shared decision-making session with a clinician. Similar to genotyping then, which determines the presence or absence of a particular gene feature, simotyping would allocate the presence or absence of a particular similarity feature, for example, a diagnosis of diabetes. In this context then, a similarity-wide association study (SiWAS) has the goal of discovering clusters of patients similar to an index patient and identifying similar features that associate with specific outcomes, such as complications, procedures, hospitalizations, or death. For example, investigating whether in patients most similar to an index patient diabetes is more likely to associate with non-healing leg ulcers, critical limb ischemia, or gangrene leading to limb amputation.

Third, patient similarity analytics have the potential to bring together a variety of omics and other systems medicine tools, if we can do so in a way that is effective, accurate, consistent, and computationally efficient (Brown, 2015a). Indeed, several groups have proposed methods of aggregating omics and monitoring these over time for individual patients, and perhaps even using comprehensive patient avatars. Integrating these methods with patient similarity has the potential to launch systems medicine further into a future where medicine is even more precisely individualized. Patient similarity will likely become and persist as a useful tool in systems medicine.

## Mathematics in patient similarity analytics

For illustration of the utility of patient similarity in medicine, only briefly presented here are a few selected examples of patient similarity analytics used for diabetes and cancer, which are common chronic or terminal diseases, respectively, currently addressed in public health. In some studies, a patient similarity metric is determined as follows (Lee et al., 2015; Li et al., 2015). A patient can be represented by a Euclidean vector. Predictor variables such as laboratory test results or vital signs can define a multi-dimensional feature space. The cosine of the angle between two patients' vectors can define the associated patient similarity metric. A dot product can facilitate the calculation. This can be termed the “cosine similarity,” defining the patient similarity metric as follows:

(1) $$\begin{array}{l} PSM\left(P_{1},P_{2}\right)=\frac{P_{1}\cdot P_{2}}{\left||P_{1}||\;||P_{2}|\right|},\\ \;=\frac{\sum_{i=1}^{n}P_{1i}\times P_{2i}}{\sqrt{\sum_{i=1}^{n}P_{1i}{}^{2}}\times \sqrt{\sum_{i=1}^{n}P_{2i}{}^{2}}} \end{array}$$

where *P*_1*i*_ and *P*_2*i*_ represent a single predictor variable vector for two separate patients, · represents the dot product, and || || represents the Euclidean vector magnitude, as shown. Since the patient similarity metric is an angle cosine, it normalizes between −1 (considered minimum possible similarity) and 1 (considered maximum possible similarity). As expected, two predictor variable vectors pointing in the exact opposite direction to each other would have a 180° angle between them, and would therefore calculate to a patient similarity metric of −1. Conversely, two perfectly overlapping vectors would have an angle of 0° between them, and would therefore calculate to a patient similarity metric of 1. Accordingly, before calculating the total patient similarity metric, the product for each predictor variable vector would be normalized to the range of −1 to 1 in the multidimensional feature space, if continuous (Lee et al., 2015). The product for categorical/binary predictor variable vectors would be assigned a value of −1 or 1. The patient similarity metric would be calculated for each patient in a given data set, relative to an index patient *P*_1_. The N most similar patients to the index patient would be utilized as a training data set for testing in a validation data set, with prediction of prognosis, morbidity, or mortality. After successful validation, the predictive model could be used for epidemiologic or clinical studies. For example, am algorithm using cosine similarity successfully identified three subgroups of patients with diabetes (Li et al., 2015). The first subgroup included patients with diabetic nephropathy (diabetes-related kidney disease) and diabetic retinopathy (diabetes-related eye disease). The second subgroup included several patients with cancer and cardiovascular diseases. The third subgroup included many patients who also had cardiovascular diseases, along with neurological diseases, allergies, and HIV infection. Various single nucleotide polymorphisms mapped to these three subgroups that were confirmed in the EHR, suggesting clinical relevance for patient similarity in precision medicine. Jaccard similarity, another metric that can be leveraged after assigning binary attributes to each patient's multifeature vector space, was useful to analyze features underlying deviant responses to therapeutics in patients with diabetes (Zhang et al., 2014).

Alternatively, unsupervised clustering of patients based on their clinical predictor variables could be used to produce a patient-patient network. The network could be organized using L-infinity centrality, which is the maximum distance from each point from any other point in a given data set. L-infinity centrality produces a detailed and succinct description of any data set yielding more information than scatter plots (Lum et al., 2013). Large values for L-infinity centrality correspond to data points at large distances from the center of the data set (Li et al., 2015). Other pattern analysis and cluster algorithms (Daemen and De Moor, 2009; Chan et al., 2010; Liu et al., 2013a; Mabotuwana et al., 2013; Sundar et al., 2014), or algorithms incorporating distance metric learning (Wang et al., 2011; Bian and Tao, 2012), locally supervised metric learning (Sun et al., 2012; Ng et al., 2015), local spline regression (Wang et al., 2012), or visual analytics (Tsymbal et al., 2009; Ebadollahi et al., 2010; Gotz et al., 2011; Perer, 2012; Heer and Perer, 2014; Bolouri et al., 2016; Ozery-Flato et al., 2016), can also be used for patient similarity to predict diabetes onset, develop treatment recommendations tailored to each patient, or predict survival after chemotherapy (Chan et al., 2010; Liu et al., 2013a; Ng et al., 2015; Ozery-Flato et al., 2016), among other applications. SNOMED CT and other medical terminology frameworks can be used to facilitate communication across platforms in various studies (Melton et al., 2006). There are also algorithms to incorporate a time series into patient similarity analysis, to predict trends over time among patients (Wu et al., 2005; Hartge et al., 2006; Ebadollahi et al., 2010; Carreiro et al., 2013; Alaa et al., 2016). For example, a patient similarity time series algorithm has been used to fine-tune radiation treatment planning for patients with head and neck cancers (Wu et al., 2005).

## Challenges in patient similarity

There are certain challenges in patient similarity, such as network bottlenecks, low hardware performance (processing power and memory), and data locality (Osman et al., 2013; Karapiperis and Verykios, 2014; Barkhordari and Niamanesh, 2015). Given the observational or retrospective nature of patient similarity, interpretation of data analysis will be imperfect. Confounder control and treatment selection bias are inherent limitations in such studies. However, groups have developed strategies to manage the potential for confounders, such as restriction, stratification, matching, inverse probability weighting, and covariate adjustment (Gallego et al., 2015). Several groups have also proposed solutions for other challenges that enable large scale patient indexing and accurate and efficient clinical data retrieval (Wang, 2015). Some have devised algorithms to address the complexity of clinical data and limited transparency of many existing clinical case retrieval decision support systems (Tsymbal et al., 2009), as well as integration of data from various heterogeneous omics studies (Wang et al., 2014, 2016; Gligorijević et al., 2016) and physician input and feedback (Wang et al., 2011; Sun et al., 2012; Fei and Sun, 2015). Others have produced algorithms that address scalability and uncertainty, by requiring parallel or distributed algorithm implementations built to scale, and enhancing interpretability by conveying the certainty of results presented (Feldman et al., 2015). One such algorithm or platform is scalable and distributable patient similarity (ScaDiPaSi), a dynamic method for investigating patient similarity that spreads the algorithm over several self-sufficient hardware nodes to process query data from various sources of different formats simultaneously (Barkhordari and Niamanesh, 2015). Another tool, MapReduce, employs several optimization techniques, such as job scheduling and cascading work flows over multiple interdependent hardware nodes (Dean and Ghemawat, 2008). Use of all of these technological solutions for patient similarity in precision medicine will be facilitated by bridging gaps among different scientific, technological, and medical cultures, through interdisciplinary collaborations among experts in medicine, biology, informatics, engineering, public health, economics, and the social sciences (Kuhn et al., 2008).

## Conclusion

Various patient similarity algorithms have been deployed and have been found beneficial by improving clinical efficiency (Wang et al., 2015), enabling secure identification of similar patients and records sharing by clinicians and rare disease scientists (Buske et al., 2015a,b), predicting patients' prognosis or trajectory over time (Ebadollahi et al., 2010; Subirats et al., 2012; Wang et al., 2012; Gallego et al., 2015), providing clinical decision support (Daemen et al., 2009; Wang et al., 2011; Subirats et al., 2012; Sun et al., 2012; Gottlieb et al., 2013; Liu et al., 2013b; Gallego et al., 2015), tailoring individual treatments (Zhang et al., 2014), preventing unexpected adverse drug reactions (Hartge et al., 2006; Yang et al., 2014), flagging patients deserving more attention due to poor response to therapies (Zhang et al., 2014; Ozery-Flato et al., 2016), and pursuing comparative effectiveness studies (Wang et al., 2011), among other applications. In general, clinical guidelines often do not supply evidence on risks, secondary therapy effects, and long-term outcomes (Gallego et al., 2015). In this setting, patient similarity analytics can provide a cheaper, portable alternative or in fact adjunct to evidence-based clinical guidelines and randomized controlled trials, particularly if trial data are unavailable for conditions or patient characteristics specific to a query individual (Longhurst et al., 2014; Gallego et al., 2015). Synthesizing current patient similarity algorithms with systems medicine tools could provide actionable insights in precision medicine.

## Author contributions

SB conceived, analyzed, designed, drafted, critically revised, approved, and agreed to be accountable for this submitted work.

### Conflict of interest statement

The authors declare that the research was conducted in the absence of any commercial or financial relationships that could be construed as a potential conflict of interest.
